# The Membrane-Associated Adaptor Protein DOK5 Is Upregulated in Systemic Sclerosis and Associated with IGFBP-5-Induced Fibrosis

**DOI:** 10.1371/journal.pone.0087754

**Published:** 2014-02-13

**Authors:** Hidekata Yasuoka, Yukie Yamaguchi, Carol A. Feghali-Bostwick

**Affiliations:** 1 Division of Pulmonary, Allergy, and Critical Care Medicine, Department of Medicine, University of Pittsburgh School of Medicine, Pittsburgh, Pennsylvania, United States of America; 2 Division of Rheumatology & Immunology, Department of Medicine, Medical University of South Carolina, Charleston, South Carolina, United States of America; 3 Division of Rheumatology, Department of Internal Medicine, Keio University School of Medicine, Tokyo, Japan; University of Texas Health Science Center at Houston, United States of America

## Abstract

Systemic sclerosis (SSc) is characterized by excessive fibrosis of the skin and internal organs due to fibroblast proliferation and excessive production of extracellular matrix (ECM). We have shown that insulin-like growth factor binding protein (IGFBP)-5 plays an important role in the development of fibrosis *in vitro, ex vivo*, and *in vivo*. We identified a membrane-associated adaptor protein, downstream of tyrosine kinase/docking protein (DOK)5, as an IGFBP-5-regulated target gene using gene expression profiling of primary fibroblasts expressing IGFBP-5. DOK5 is a tyrosine kinase substrate associated with intracellular signaling. Our objective was to determine the role of DOK5 in the pathogenesis of SSc and specifically in IGFBP-5-induced fibrosis. DOK5 mRNA and protein levels were increased *in vitro* by endogenous and exogenous IGFBP-5 in primary human fibroblasts. DOK5 upregulation required activation of the mitogen-activated protein kinase (MAPK) signaling cascade. Further, IGFBP-5 triggered nuclear translocation of DOK5. DOK5 protein levels were also increased *in vivo* in mouse skin and lung by IGFBP-5. To determine the effect of DOK5 on fibrosis, DOK5 was expressed *ex vivo* in human skin in organ culture. Expression of DOK5 in human skin resulted in a significant increase in dermal thickness. Lastly, levels of DOK5 were compared in primary fibroblasts and lung tissues of patients with SSc and healthy donors. Both DOK5 mRNA and protein levels were significantly increased in fibroblasts and skin tissues of patients with SSc compared with those of healthy controls, as well as in lung tissues of SSc patients. Our findings suggest that IGFBP-5 induces its pro-fibrotic effects, at least in part, via DOK5. Furthermore, IGFBP-5 and DOK5 are both increased in SSc fibroblasts and tissues and may thus be acting in concert to promote fibrosis.

## Introduction

Systemic sclerosis (SSc) is characterized by excessive organ fibrosis due to fibroblast proliferation and production of extracellular matrix (ECM) [1]. Our group previously reported increased expression of insulin-like growth factor binding protein (IGFBP)-5 in primary early-passage dermal fibroblasts cultured from patients with SSc [2]. We also reported that IGFBP-5 mRNA and protein levels are increased *in vivo* in lung tissues of patients with idiopathic pulmonary fibrosis (IPF) and *in vitro* in primary fibroblasts cultured lung tissues of patients with SSc and those with IPF [3], [4]. Interestingly, IGFBP-5 triggers a fibrotic phenotype *in vitro* [3] and *in vivo* [4], [5] that includes induction of ECM production, myofibroblastic transformation and infiltration of mononuclear cells [6]. Furthermore, IGFBP-5 induces dermal fibrosis in human skin maintained in organ culture [7]. These findings suggest that IGFBP-5 is responsible, at least in part, for the fibrosis characteristic of SSc.

We used gene expression profiling of primary fibroblasts expressing IGFBP-5 to identify downstream effectors of IGFBP-5 and found a membrane-associated adaptor protein, downstream of tyrosine kinase/docking protein (DOK)5, as an IGFBP-5 target gene. DOK5 belongs to the docking family of proteins that contain tandem pleckstrin homology-phosphotyrosine binding (PH-PTB) domains at their amino termini [8]. DOK proteins are also known as insulin receptor substrates (IRS) and serve as substrates for insulin and insulin-like growth factor [9] as well as various protein tyrosine kinases [10], [11]. DOK5 functions as a specific signal transduction molecule and plays an important role in cellular differentiation [11], [12]. Our goal was to assess the levels of DOK5 in fibrotic tissues from patients with SSc and to delineate the role of DOK5 in IGFBP-5-induced fibrosis.

## Materials and Methods

### Lung and Skin Samples

Human skin was obtained from corrective plastic surgery and maintained in organ culture as we have previously reported [7], [13]. Skin punch biopsies were obtained from the clinically affected and unaffected skin of patients with SSc as we have previously described [2]. Lung tissues were obtained from the explanted lungs of patients with SSc-associated pulmonary fibrosis who underwent lung transplantation at the University of Pittsburgh Medical Center, and normal donors whose lungs were not used for transplantation as previously reported [14], [15]. All tissues were obtained under a protocol approved by the Institutional Review Board of the University of Pittsburgh and following written informed consent. Relevant information on the patients with SSc is summarized in Tables 1 & 2.

**Table 1:** Description of SSc patients from whom skin biopsies were obtained.

Patient code	Age (yrs)	Gender	Disease duration(yrs)	Disease type	Auto-antibody	Treatment (at the time of biopsy)
SSc-101	41.8	Male	8.1	Diffuse	RNA pol, PL-12	Prednisone, Penicillamine, Methotrexate
SSc-102	38.6	Female	0.7	Diffuse	RNA pol	None
SSc-103	67.2	Female	0.9	Diffuse	N/A	Methotrexate

**Table 2:** Description of SSc patients from whom lung tissues were obtained.

Lung Sample	Age (yrs)	Gender	Histology
SSc-24	45.1	Male	UIP/NSIP
SSc-26	57.5	Male	UIP
SSc-30	58.8	Female	UIP

### Primary Fibroblast Culture

Human primary lung and skin fibroblasts were cultured from the explanted lungs of normal organ donors or the skin of healthy controls under a protocol approved by the University of Pittsburgh Institutional Review Board. Primary fibroblasts were also cultured from the skin of twins discordant for SSc. Approximately 2–3 mm^2^ pieces of tissue were minced and fibroblasts were cultured in Dulbecco’s modified Eagle medium (DMEM) supplemented with 10% fetal bovine serum (FBS), penicillin, streptomycin, and anti-mycotic agent, as previously described [3]. All cells were used between passages 4–6.

### Adenovirus Construct Preparation

Adenovirus constructs were obtained as previously reported [3]. Briefly, the full-length cDNAs of human DOK5, IGFBP-3 and IGFBP-5 were obtained by reverse transcriptase-polymerase chain reaction (RT-PCR) using total RNA extracted from primary human fibroblasts. The cDNAs were subcloned into the shuttle vector pAdlox and used for the preparation of replication-deficient adenovirus serotype 5 expressing DOK5 (DOK5 AdV), IGFBP-3 (IGFBP-3 AdV), IGFBP-5 (IGFBP-5 AdV), or no cDNA (Control AdV) in the Vector Core Facility of the University of Pittsburgh.

### In Vivo Experiments

Administration of adenovirus to mice was done as previously described [4], [5]. All studies and procedures were approved by the University of Pittsburgh Institutional Animal Care and Use Committee. All efforts were made to minimize suffering. Wild-type C57BL/6J mice were intratracheally or intradermally injected with Control AdV, DOK5 AdV, IGFBP-5 AdV or IGFBP-3 AdV. Mice were sacrificed 8 days post adenoviral administration. Harvested lung and skin tissues were fixed with 10% formalin and embedded in paraffin.

### Preparation of Nuclear and Cytoplasmic Extracts

Nuclear and cytoplasmic extracts were prepared as previously reported [16]. Briefly, 1.2×10^6^ fibroblasts were cultured on 10-cm dishes and scraped with Buffer A (10 mM HEPES-KOH pH 7.9, 1.5 mM MgCl_2_, 10 mM KCl, 0.5 mM DTT, 0.2 mM PMSF). Lysates were incubated on ice for 10 minutes. After centrifugation at 12,000 rpm for 30 seconds, supernatant (cytoplasmic extract) was harvested. The pellet was resuspended in Buffer C (20 mM HEPES-KOH pH7.9, 25% glycerol, 420 mM NaCl, 1.5 mM MgCl_2_, 0.2 mM EDTA, 0.5 mM DTT, 0.2 mM PMSF) and incubated on ice for 20 minutes. After centrifugation at 12,000 rpm for 30 seconds, nuclear extracts were harvested and used for immunoblotting.

### Preparation of Lung Homogenates

Lung tissues were weighed and homogenized with a tissue homogenizer in 10 volumes of RIPA buffer with protease inhibitor and 0.5% Triton-X. Following centrifugation (13,000×g at 4°C for 30 minutes), the supernatant was collected. Samples were mixed with sample buffer containing SDS and used for immunoblotting.

### Immunohistochemistry and Immunocytostaining

For immunohistochemistry, sections of paraffin-embedded lung tissues were de-paraffinized, and endogenous peroxidase and biotin were quenched. For immunocytostaining, fibroblasts were cultured on cover slips coated with type I collagen. Sections or cover glasses were blocked with 5% serum and incubated with anti-DOK5 antibody followed by secondary antibody. Bound secondary antibody was detected using the AEC Red kit or Fluorescence detection kit. A light hematoxylin counterstain (immunohistochemistry) and DAPI or TO-PRO-1 (Molecular Probes, Eugene, OR) was used to identify nuclei. Images were taken on a Nikon Eclipse 800 microscope or Olympus Fluoview 1000 using identical camera settings. Intensity of signal was examined using MetaMorph® software.

### Immunoblotting

Fibroblasts were cultured in medium supplemented with 500 ng/ml recombinant human IGFBP-5 or 5μl 10 mM HCl as a vehicle control. For adenoviral infection, fibroblasts were transduced with Control AdV, DOK5 AdV, IGFBP-5 AdV or IGFBP-3 AdV at an MOI of 50. Culture supernatants and cellular lysates were harvested and analyzed by immunoblotting. Signals were detected following incubation with horseradish peroxidase-conjugated secondary antibody and chemiluminescence.

### Detection of DOK5 mRNA

DOK5 and β-actin mRNA expression in cultured fibroblasts was detected using RT-PCR. Primer sets were forward: 5′-CACGGATCAATGACATCAGC-3′, reverse: 5′-TCAGAGGGCTGGAAACATCT-3′ to amplify DOK5 (534 bp) and forward: 5′-ATGTTTGAGACCTTCAACAC-3′, reverse: 5′-CACGTCACACTTCATGATGG-3′ to amplify β-actin (494 bp). PCR conditions were 3 min at 94°C, followed by 35 (for DOK5) or 30 (for β-actin) cycles of 1 min at 94°C, 1 min at 60°C, and 1 min at 72°C. PCR products were separated by electrophoresis on agarose gels and stained with ethidium bromide.

### Ex Vivo Human Skin Culture

Human skin culture was done as previously reported [7], [13]. Briefly, skin tissue obtained from plastic surgery was cut into 1.5 cm×1.5 cm sections and subcutaneous fat was trimmed. 1×10^8^ pfu AdV were injected intradermally. Dermal layers were cultured in an air liquid interface with the epidermal layer side up and exposed to air. DMEM supplemented with 10% FBS, penicillin, streptomycin, and anti-mycotic agent was replaced daily. Skin tissue was harvested and fixed in 10% formalin prior to embedding in paraffin.

### Statistical Analysis

Statistical comparisons were performed using the Mann-Whitney U-test or one-way ANOVA as appropriate.

## Results

### DOK5 Expression is Upregulated by Endogenous and Exogenous IGFBP-5 via MAPK

**Figure 1:**
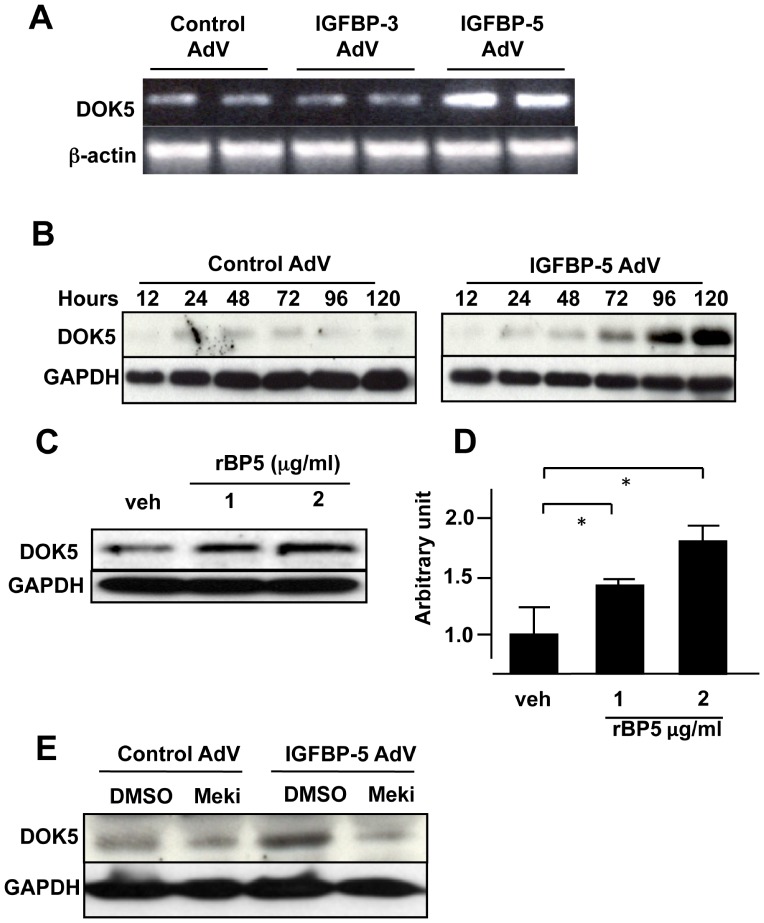
DOK5 expression is upregulated by endogenous and exogenous IGFBP-5 via MAPK. A. DOK5 mRNA is upregulated by endogenous IGFBP-5. Human skin fibroblasts were infected with adenovirus expressing IGFBP-3, IGFBP-5 or control (IGFBP-3 AdV, IGFBP-5 AdV, or control AdV, respectively) at an MOI of 50. Expression of DOK5 was examined by RT-PCR. β-actin is shown as a loading control. Results are representative of two independent experiments using different fibroblast strains. B. Production of DOK5 protein is induced by endogenous IGFBP-5. Production of DOK5 was examined by immunoblotting in whole cell lysates treated as in A. Molecular weight of DOK5 is ∼30 kDa. GAPDH is shown as a loading control. The experiment was repeated twice with reproducible results. C. Production of DOK5 protein is induced by exogenous IGFBP-5. Human skin fibroblasts were stimulated with recombinant IGFBP-5 (rBP5), and whole cell lysates were harvested. DOK5 production was examined by immunoblotting. GAPDH is shown as a loading control. The experiment was repeated three times. D. Graphical presentation of the data shown in panel C. *indicates p<0.05. E. Induction of DOK5 protein production is blocked by the Mek inhibitor (MEKi). Meki was administered after infection with AdVs and levels of DOK5 examined by immunoblotting. GAPDH is shown as a loading control. Data shown are representative of two independent experiments.

To delineate the pathways involved in IGFBP-5-induced fibrosis, we examined the effect of IGFBP-5 on DOK5 levels. As shown in Figure 1A and B, both DOK5 mRNA and protein levels are increased in IGFBP-5-expressing human skin fibroblasts compared with control AdV-treated fibroblasts. Exogenous administration of physiological concentrations of IGFBP-5 also induced the expression of DOK5 (Figure 1C and D). DOK5 upregulation was regulated by MAPK activation since the MEK inhibitor U0126 abrogated IGFBP-5 induction of DOK5 (Figure 1E).

### DOK5 Localizes to the Nucleus

**Figure 2:**
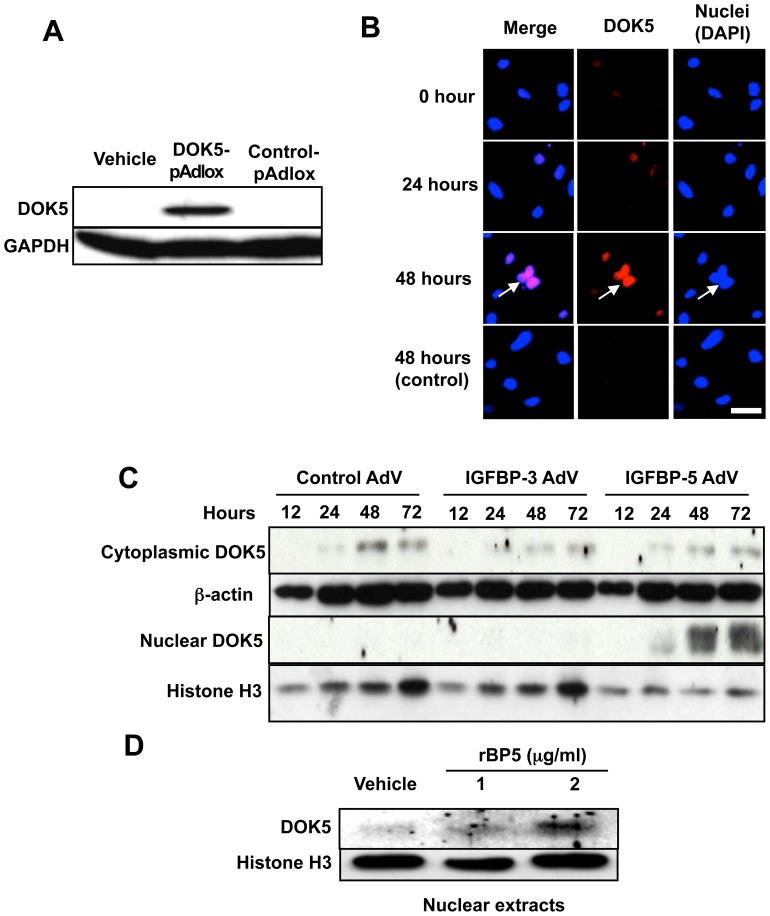
DOK5 localizes to the nucleus. A. Confirmation of DOK5 expression. Expression of DOK5 was confirmed by immunoblotting following transient transfection of COS7 cells with DOK5-pAdlox or control-pAdlox plasmids. GAPDH is shown as a loading control. Data were confirmed in an additional two experiments. B. DOK5 translocates to the nucleus. DOK5 localization in COS7 cells treated as in A using immunofluorescence. DOK5 is shown in red and nuclei stained with DAPI appear blue. Merged signal appears purple. Indicated times reflect hours post-transfection. Magnification, 800x. C. Nuclear DOK5 levels are increased by endogenous IGFBP-5. Lysates of primary human fibroblasts infected with adenovirus (AdV) were fractionated into cytoplasmic and nuclear fractions and induction of DOK5 was examined using immunoblotting. β-actin and Histone H3 are shown as loading control for cytoplasmic and nuclear fractions, respectively. Findings were confirmed in two independent experiments. D. Nuclear DOK5 levels are induced by exogenous IGFBP-5. Cytoplasmic fractions of primary human fibroblasts treated with recombinant IGFBP-5 (rBP5) were harvested and induction of DOK5 was examined. Histone H3 was used as a loading control. Findings were confirmed in two independent experiments.

To identify the intracellular localization of DOK5, we detected DOK5 following its overexpression as well as its induction by IGFBP-5. A DOK5-expressing pAdlox construct was generated and DOK5 expression confirmed via transient transfection of COS7 cells with DOK5-pAdlox vector (Figure 2A). Using this vector, intracellular localization was examined by immunocytostaining. DOK5 localized to cell nuclei (Figure 2B) suggesting that the vector-encoded DOK5 retained its ability to translocate to the nucleus. We then examined intracellular localization of DOK5 following induction by IGFBP-5 in cytoplasmic and nuclear extracts of cultured primary human skin fibroblasts infected with IGFBP-5 AdV, IGFBP-3 AdV as a related control protein or control AdV. DOK5 was detectable in cytoplasmic fractions under all treatment conditions, but DOK5 levels were only increased in the nuclear fraction (Figure 2C) of IGFBP-5 expressing fibroblasts. Similar results were obtained in fibroblasts treated with recombinant IGFBP-5 (Figure 2D). Thus, both endogenous and exogenous IGFBP-5 induce DOK5 expression and nuclear localization.

### DOK5 Production is Increased In Vivo by IGFBP-5

**Figure 3:**
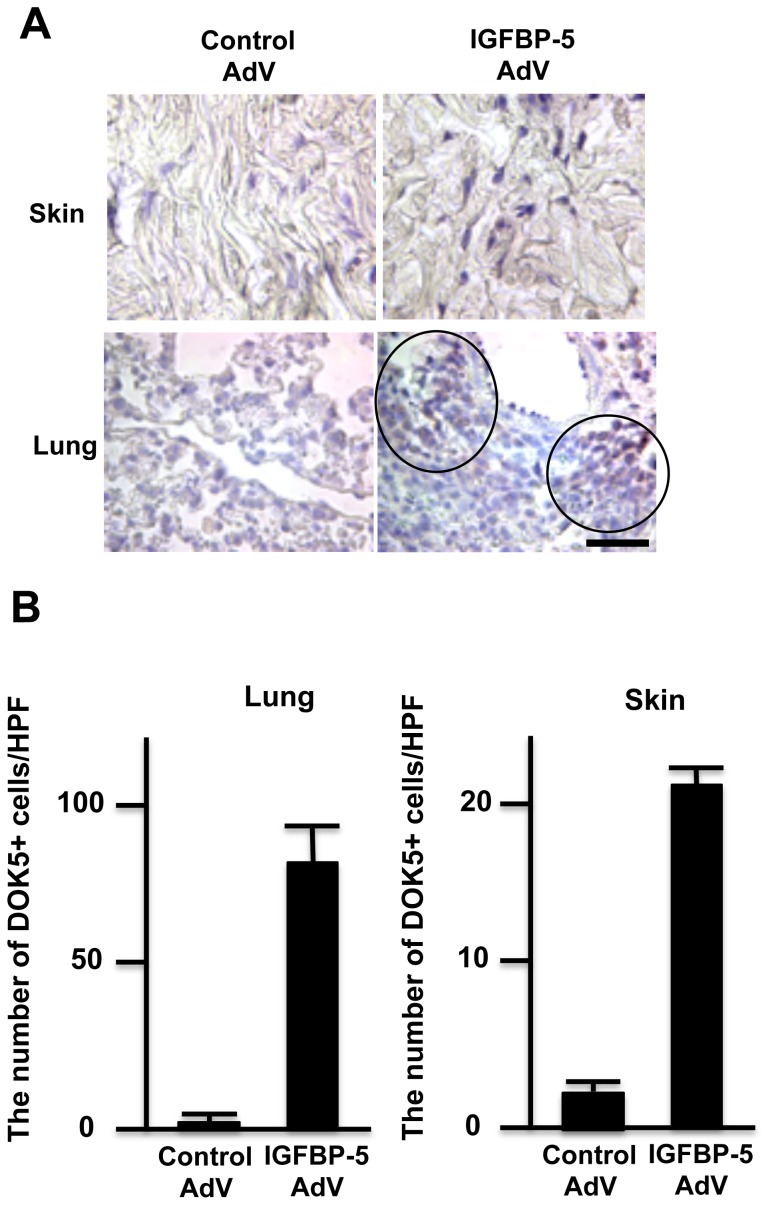
DOK5 is upregulated in lung and skin tissues engineered to express IGFBP-5 and localizes to cell nuclei *in vivo*. **A.** DOK5 expression was examined in skin (left panel) and lung (right panel) tissues of C57BL6/J mice treated with control or IGFBP-5 expressing adenovirus (control AdV and IGFBP-5 AdV, respectively). DOK5-expressing cells appear brown following AEC staining. Magnification, 800x. Data are representative of skin and lung tissues from 2 mice/group. **B.** Graphical presentation of data shown in A using 8 fields per treatment condition. Y axis shows the number of DOK5-positive cells counted at a magnification of 400X. Bars denote the mean and error bars denote SD.

To determine whether IGFBP-5 also induces DOK5 *in vivo*, IGFBP-5 AdV or control AdV, were administered to mouse lung or skin and DOK5 detected using IHC. DOK5 localized to cellular nuclei in skin and lung mouse tissues engineered to express IGFBP-5 as shown in Figure 3A. Based on morphology, DOK5 was detectable in fibroblasts in skin tissues, and in cellular infiltrates such as mononuclear cells and in epithelial cells in lung tissues. The number of DOK5 positive cells was increased by IGFBP-5 in both skin and lung (Figure 3B).

### DOK5 Induces a Fibrotic Phenotype Ex Vivo in Human Skin

**Figure 4:**
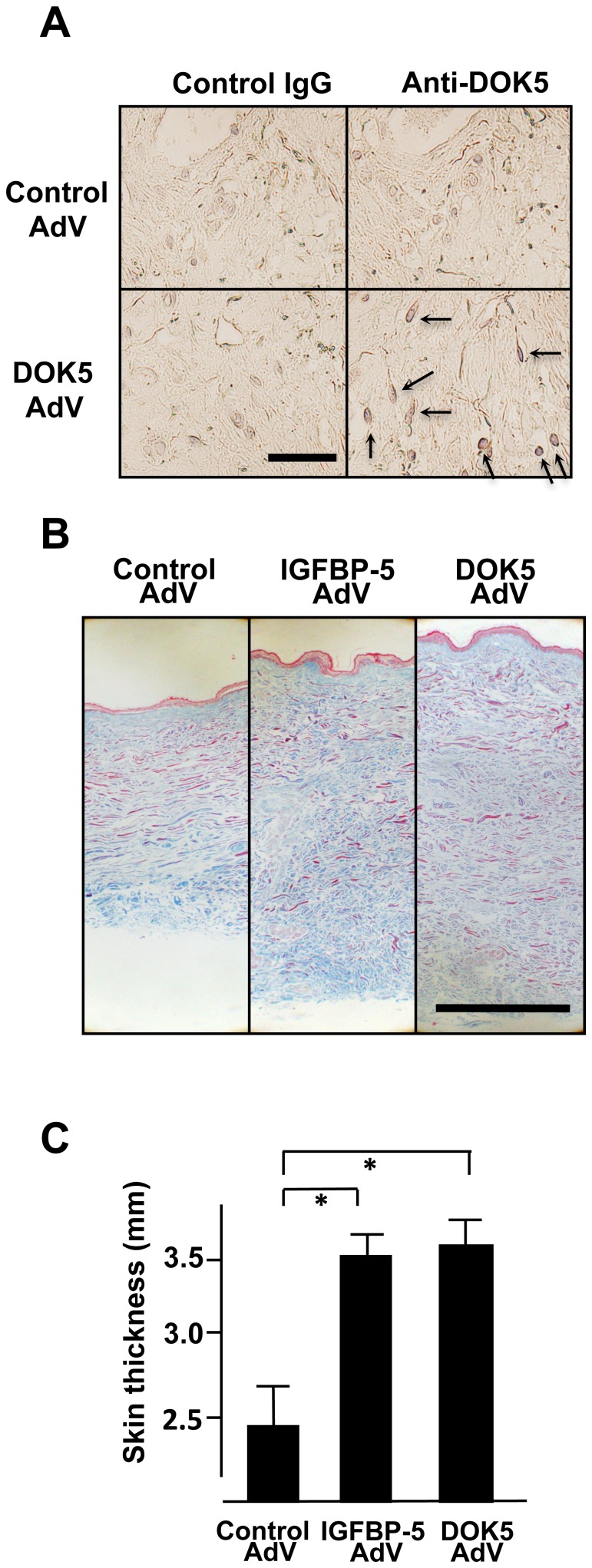
DOK5 expression increases dermal thickness. A. Representative results of organ culture of human skin engineered to express IGFBP-5, DOK5, or control. DOK5 expression was examined in human skin treated with control or IGFBP-5 expressing adenovirus (control AdV and IGFBP-5 AdV, respectively). DOK5-expressing cells were shown in red using AEC staining. Magnification, 800x. Results are representative of three experiments. B. DOK5 triggers dermal fibrosis. Dermal thickness and collagen content as assessed by Masson Trichrome are increased in DOK5-treated human skin. Magnification, 40x. Findings were replicated using skin from two different donors. C. DOK5 induces a significant increase in dermal thickness. Dermal thickness was measured in human skin and represents the average of 4 experiments. Values shown represent mean and SD. *P<0.008.

We examined whether DOK5 can directly induce a fibrotic phenotype in a human tissue and thus serve as an intermediate for IGFBP-5. We used human skin engineered to express DOK5 or IGFBP-5 using DOK5 AdV and IGFBP-5 AdV, respectively [7]. As shown in Figure 4A, DOK5 is expressed in dermal fibroblasts. We then examined the ability of DOK5 to induce collagen deposition and dermal thickening using Masson Trichrome staining (Figure 4B). DOK5 induced significant skin thickening similarly to IGFBP-5 (Figure 4C) [7]. These results suggest that DOK5 can directly induce fibrosis in human skin.

### DOK5 Protein Levels are Increased in Lung and Skin Tissues of SSc Patients

**Figure 5:**
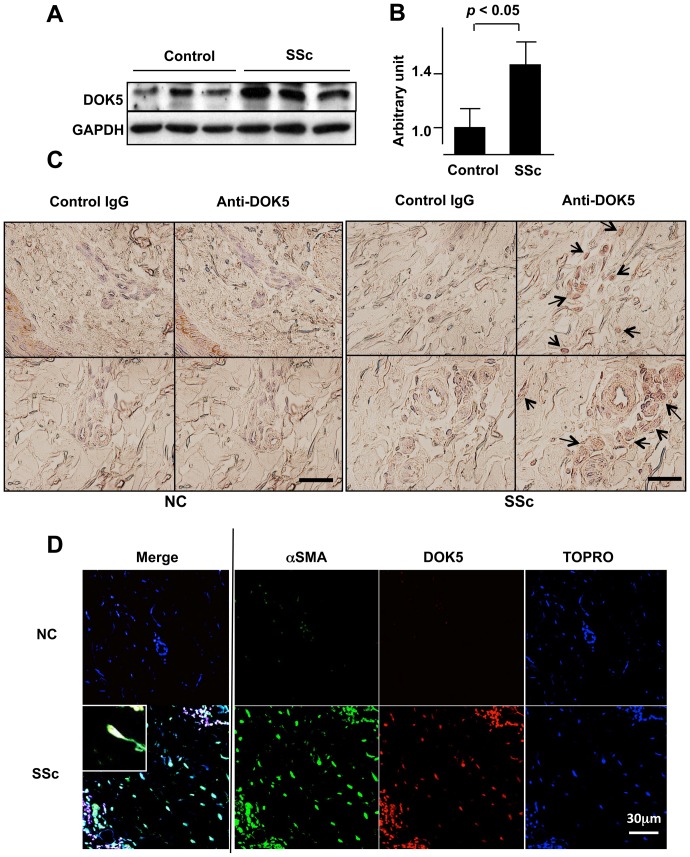
DOK5 mRNA and protein levels are increased in lung and skin tissues of SSc patients. A. DOK5 protein is increased in lung tissues of SSc patients. DOK5 protein was detected in total lung tissue homogenates of patients with SSc and healthy controls using immunoblotting. B. DOK5 protein levels are significantly elevated in SSc lung tissues. Data presented show average signal intensity of DOK5 in lung homogenates from 3 patients with SSc and 3 healthy controls. Protein levels were examined using immunoblotting and signals quantified with densitometry. *P<0.05. C. Representative images of DOK5 in skin tissue from a patient with SSc and a normal control (NC). DOK5 was detected using immunohistochemistry. Brown signal indicates DOK5. Arrows point to representative cells positive for DOK5. Sections were counter-stained with hematoxylin. Images are representative of sections from three patients and three controls. Bar = 20 µm. Magnification, 400x. D. Localization of DOK5 and αSMA in SSc skin. White color indicates merging of blue, red and green, and shows expression of DOK5 in nuclei. Images are representative of immunofluorescence staining done on skin samples from two patients with SSc and two controls. Bar = 20 µm.

We previously reported increased IGFBP-5 in fibrotic lung and skin of patients with SSc and lung tissues of patients with IPF [2]–[4] and that IGFBP-5 can induce fibrosis *in vitro* and *in vivo* [3], [4]. Since DOK5 is induced by IGFBP-5, we hypothesized that DOK5 may be increased in SSc tissues and primary fibroblasts. DOK5 was detected in lung homogenates by immunoblotting (Figure 5A) and its levels were significantly higher in lung tissues of SSc patients compared to those from normal controls (Figure 5B). In addition to fibrotic lung tissues of patients with SSc, increased DOK5 levels were also noted in SSc skin, where DOK5 was detected in fibroblasts, endothelial cells, and some infiltrating immune cells (Figure 5C).

Co-localization of DOK5 and αSMA was examined. As shown in Figure 5D, DOK5 was expressed in αSMA-expressing fibroblasts and infiltrating mononuclear cells surrounding blood vessels. DOK5 was detected in cell nuclei *in vivo* (inset).

**Figure 6:**
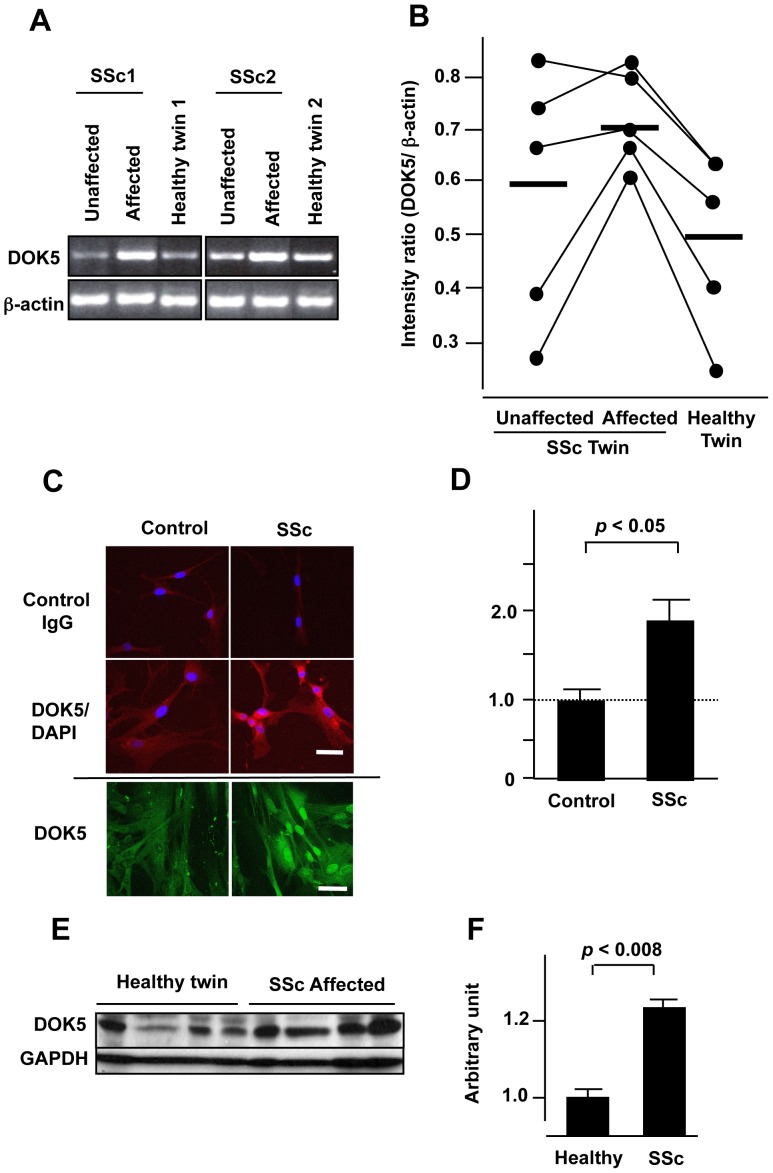
DOK5 mRNA and protein levels are increased in cultured dermal fibroblasts of patients with SSc. A. DOK5 mRNA levels are increased in dermal fibroblasts of patients with SSc compared to fibroblasts from their healthy twin. Expression of DOK5 was examined using RT-PCR. Representative results of samples from 2 pairs of identical twins discordant for SSc are shown. B. DOK5 mRNA levels are significantly higher in SSc fibroblasts. Graphical presentation is from data obtained from dermal fibroblasts of 5 pairs of twins discordant for SSc. *P*<0.05 by one-way ANOVA. C. Representative results of DOK5 protein detected by immunofluorescence in control and SSc fibroblasts. Magnification, 400x. D. DOK5 protein levels are significantly increased in SSc dermal fibroblasts. Levels of DOK5 protein in dermal fibroblasts from 3 controls and 3 patients with SSc were detected by immunocytochemistry and fluorescence intensity measured using Metamorph. *P<0.05. E. DOK5 protein levels are increased in SSc fibroblasts. DOK5 proteins were detected by immunoblotting in skin fibroblasts of 4 twins discordant for SSc. F. DOK5 protein levels are significantly higher in SSc fibroblasts. Graphical presentation of immunoblotting results shown in panel E using fibroblasts from 4 patients with SSc and their healthy twins. *P<0.008. The twin cohort was previously described [22].

Increased DOK5 expression in SSc dermal fibroblasts was confirmed using skin fibroblasts from twins discordant for SSc. As shown in Figure 6A and B, DOK5 mRNA was increased in fibroblasts from the clinically affected skin of the SSc patients compared with their own uninvolved skin fibroblasts or those of their healthy twin. DOK5 protein levels were also significantly increased in SSc dermal fibroblasts compared with fibroblasts from the patients’ healthy twins when detected using immunocytochemistry (Figure 6C and D) or immunoblotting of cell homogenates (Figure 6E and F).

## Discussion

We previously reported increased expression of IGFBP-5 in primary dermal fibroblasts of patients with SSc [2], and increased levels of IGFBP-5 mRNA and protein in lung tissues of patients with IPF and SSc [3] and in primary fibroblasts cultured from these tissues [3], [4]. Interestingly, IGFBP-5 induces ECM production and fibrosis *in vitro* [3] *in vivo* [4], [5], and *ex vivo* [7]. In this study, we identified a new signaling molecule, DOK5, downstream of IGFBP-5-induced fibrosis. We showed that DOK5 was upregulated by endogenous and exogenous IGFBP-5 stimulation via MAPK signaling, and expression of DOK5 increased dermal thickness of human skin similarly to IGFBP-5. Also, DOK5 levels were increased in cultured fibroblasts and in myofibroblasts in tissues of patients with SSc. Thus, these results suggest that upregulated expression of DOK5 is also associated with the development of fibrosis and the pathogenesis of SSc.

DOK5 is one of seven proteins in the mammalian Dok protein family [8]. The Dok proteins are structurally similar with conserved amino-terminal pleckstrin homology and phosphotyrosine-binding domains followed by Src homology 2 (SH2) target motifs in the COOH-terminal region, suggesting they may have adaptor function. Since they can also recruit and promote the assembly of specific signal transduction molecules via these motifs, DOK proteins may play an important role in the transduction of intracellular signals. Moreover, DOK proteins are also known as insulin receptor substrates, and serve as substrates for various protein tyrosine kinases. For example, DOK5 is reported as a substrate for the c-Ret receptor tyrosine kinase [11] and tropomyosin-related kinase (Trk) family receptors (TrkB/C) [10]. Functionally, DOK5 is involved in neuronal [11] and cardiomyocyte differentiation [12]. We show that DOK5 also induces a fibrotic phenotype. Since DOK4, 5, and 6 are preferentially expressed in non-hematopoietic cells as signal activation adaptors [10], [11], whereas DOK1, 2, and 3 are mainly expressed in hematopoietic cells as signal inhibitors [8], our results suggest that DOK5 promotes fibrotic signals in non-hematopoietic cells such as fibroblasts. DOK5-induced fibrosis was a little more pronounced than that of IGFBP-5. The effect of the two proteins is not significantly different and may result from a potentially more efficient adenoviral infection with the DOK5 Adv. Another potential explanation is that IGFBP-5 likely induces expression of mediators of fibrosis such as DOK5 as well as components of a regulatory network that dampen its effects, whereas DOK5 expression may not induce the same negative feedback pathways as IGFBP-5 and/or may not activate them to the same extent.

As shown in this study, induction of DOK5 by IGFBP-5 requires extracellular MAPK activation. We previously reported that early growth response protein-1 (Egr-1) was also upregulated by IGFBP-5 via MAPK signaling, and MAPK activation mediated the induction of ECM production [14]. Moreover, this effect was independent of IGF-I, suggesting a direct effect of IGFBP-5. Our current results suggest that increased production of DOK5 is regulated in a similar manner to Egr-1. DOK5 is also reported as a substrate for insulin and IGF receptors [9]. Serum IGF-I levels are elevated in patients with diffuse cutaneous SSc compared with limited cutaneous SSc, and also in patients with pulmonary fibrosis [17]. Furthermore, both IGF-I [18] and IGF-II [19] enhance collagen synthesis in fibroblasts leading to fibrosis. Thus, it is possible that upregulated DOK5 in SSc fibroblasts also contributes to IGF-mediated induction of fibrosis. It is also conceivable that upregulated expression of DOK5 contributes to the development of a fibrotic phenotype directly downstream of IGFBP-5 and possibly other members of the IGF family.

Interestingly, DOK5 was described as a membrane-associated adaptor protein [20]. However, in our study, DOK5 induced by IGFBP-5 translocated to the nucleus. Also, DOK5 was in the lipid raft fractions of IGFBP-5-expressing fibroblasts (data not shown). Since we previously reported that IGFBP-5 translocates from the plasma membrane to the nucleus via caveolae [21], it is likely that DOK5 translocates with IGFBP-5 to the nuclear compartment. This is further supported by a report showing DOK5 to be involved in the translocation of Foxa1 from the nucleus to the cytoplasm [12]. Our findings suggest that IGFBP-5 stimulation directs DOK5 translocation from the cytoplasm to the nucleus.

In summary, our findings show that IGFBP-5 induces its pro-fibrotic effects, at least in part, via DOK5. Furthermore, IGFBP-5 and DOK5 are both increased in SSc fibroblasts and tissues, and may thus be acting in concert to promote fibrosis. DOK5 may be a novel regulator of ECM production and can exert its effects independently of IGFBP-5.
